# Insoluble Fiber in Young Barley Leaf Suppresses the Increment of Postprandial Blood Glucose Level by Increasing the Digesta Viscosity

**DOI:** 10.1155/2013/137871

**Published:** 2013-11-24

**Authors:** Akira Takano, Tomoyasu Kamiya, Hiroshi Tomozawa, Shiori Ueno, Masahito Tsubata, Motoya Ikeguchi, Kinya Takagaki, Ayaka Okushima, Yu Miyata, Shizuka Tamaru, Kazunari Tanaka, Toru Takahashi

**Affiliations:** ^1^Research and Development Division, Toyo Shinyaku Co. Ltd., 7-28 Yayoigaoka, Tosu-shi, Saga 841-0005, Japan; ^2^Graduate School of Human Health Science, University of Nagasaki, 1-1-1 Manabino, Nagayo-cho, Nishisonogi-gun, Nagasaki 851-2195, Japan; ^3^Department of Nutritional Science, Faculty of Nursing and Nutrition, University of Nagasaki, 1-1-1 Manabino, Nagayo-cho, Nishisonogi-gun, Nagasaki 851-2195, Japan; ^4^Graduate School of Human Environment Science, Fukuoka Women's University, 1-1-1 Kasumigaoka, Higashi-ku, Fukuoka 813-0003, Japan

## Abstract

Barley (*Hordeum vulgare* L.) is a well-known cereal plant. Young barley leaf is consumed as a popular green-colored drink, which is named “Aojiru” in Japan. We examined the effects of barley leaf powder (BLP) and insoluble fibers derived from BLP on postprandial blood glucose in rats and healthy Japanese volunteers. BLP and insoluble fibers derived from BLP suppressed the increment of postprandial blood glucose levels in rats (*P* < 0.01), and increased the viscosity of their digesta. The insoluble fibers present in BLP might play a role in controlling blood glucose level by increasing digesta viscosity. In human, BLP suppressed the increment of postprandial blood glucose level only in those which exhibited higher blood glucose levels after meals (*P* < 0.01). BLP might suppress the increment of postprandial blood glucose level by increasing digesta viscosity in both of rats and humans who require blood glucose monitoring.

## 1. Introduction

Barley (*Hordeum vulgare* L.) is a well-known cereal plant, which has been cultivated since ancient times as feed for livestock [1, 2]. In Japan, Korea, and China, barley is used in foods such as bread and cakes [3]. The consumption of a green-colored drink made from the powder of young barley leaves named “Aojiru” is also prevalent in Japan [1]. Recently, several animal studies have reported salutary effects accompanying the intake of barley leaves, including antiulcer, antioxidant, hypolipidemic, antidepressant, and antidiabetic effects [4–8]. Thus, young barley leaves or derivatives such as “Aojiru” might serve as functional foods or supplements to support human health.

Young barley leaf is rich in dietary insoluble fiber. Insoluble fibers have various functions [9–11] and are known to increase digesta viscosity in rats [9]. Previously, high digesta viscosity due to insoluble fibers was observed to suppress the increment of postprandial blood glucose level in rats, by decreasing the self-diffusion of glucose in digesta in the lumen [11]. Hence, the insoluble fibers in young barley leaves might serve to control blood glucose levels, as indicated in that study [11]. However, young barley leaves also possess polyphenols, including flavonoids, which are well-known antioxidants that prevent various diseases [2, 12]. Notably, lutonarin is the major compound in water extracts of young barley leaves [1]. The water soluble substances in young barley leaves may also possess functions regarding blood glucose. 

In the present study, we used barley leaf powder (BLP), which is composed of young barley leaves via the harvesting of young barley leaves, followed by washing, drying, and powdering, and examined the effects of BLP and the insoluble fibers in BLP on postprandial blood glucose in rats and healthy Japanese volunteers.

## 2. Materials and Methods

### 2.1. Materials

Barley leaf powder (BLP) was supplied by Toyo Shinyaku Co., Ltd. (Saga, Japan). BLP is produced from the young leaf of *Hordeum vulgare* L. harvested before sprouting (the height: between 300 and 600 mm), by washing, drying, and powdering. The color of BLP ranges from light to dark-green. Unless otherwise noted, all employed chemicals were of the purest grade and were obtained from Wako Pure Chemical Industries, Ltd. (Osaka, Japan). 

### 2.2. Preparation of Soluble and Insoluble Fractions

**Figure 1:**
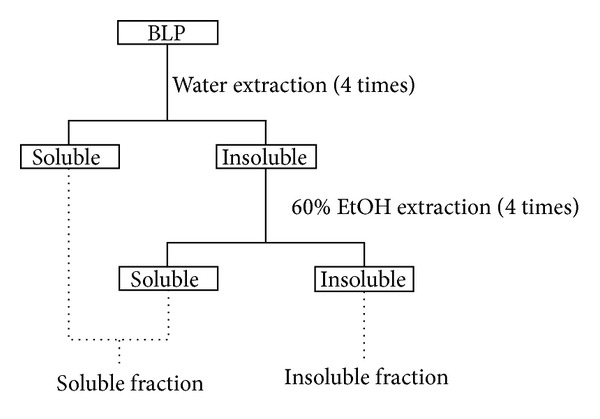
Preparation flow for the water-EtOH soluble and insoluble fractions of BLP.

Young barley leaves are rich in insoluble fiber and flavonoids, such as saponarin and lutonarin, which are soluble in water and EtOH. In order to separate insoluble fiber from flavonoids, insoluble and soluble fractions were prepared from BLP via water and ethanol extraction, with sonication and immersion (Figure 1 shows a detailed process flow). Twenty grams of BLP was suspended in 200 mL of distilled water. Sonication of this suspension followed by overnight immersion of the BLP at 4°C was performed to extract the water-soluble fraction in water. After centrifugation (10,000 g, 10 min, 4°C), the precipitant was resuspended in 200 mL of distilled water. This suspension was then sonicated for 10 minutes and immersed for 1 hour at room temperature. This sonication followed by 1 hour immersion was repeated 4 times. After centrifugation, the precipitant was resuspended in 200 mL of 60% ethanol. This ethanol suspension was then sonicated for 10 minutes and the precipitant was immersed overnight at 4°C. After centrifugation, the precipitant was resuspended in 200 mL of 60% ethanol, sonicated, and immersed for 1 hour at room temperature. This sonication followed by 1 hour immersion was repeated 4 times, using a 60% ethanol solvent. All supernatants were concentrated via evaporation. After 60% ethanol extraction, the precipitant was freeze dried using vacuum freeze-drying equipment (FDU-2100, Tokyo Rikakikai Co., Ltd.) and employed as the BLP insoluble fraction. The evaporated samples from the water and 60% ethanol extractions were mixed. This mixture was freeze dried using vacuum freeze-drying equipment and employed as the BLP soluble fraction. These fractions were used to elucidate the effect of BLP on postprandial blood glucose, yielding the results shown in Figure 3. The dietary fiber composition of BLP and these fractions are described in Table 1.

### 2.3. Determination of Total Polyphenols

Total polyphenols in 0.5 grams of BLP, the insoluble fraction, and the soluble fraction were analyzed by Folin-Denis methods (reference standard; chlorogenic acid, Sigma-Aldrich Co. LLC.) after ultrasound extraction for 15 minutes using 70% methanol.

### 2.4. Preparation of the Insoluble Fiber Rich Fraction

 Unfortunately, the content of the insoluble fiber in the “insoluble fraction” (see Section 2.2) was not particularly high (Table 1). Accordingly, we prepared a new fraction, named “insoluble fiber rich fraction in BLP”, from BLP to increase the insoluble fiber content. The insoluble fiber rich fraction in BLP was prepared using modified EFRF methods [13]. Fifty grams of BLP was suspended in 500 mL of 0.1 N HCl containing 5 g of pepsin. This suspension was incubated for 3 hours at 37°C, followed by neutralization and heating for 10 minutes in a boiling water bath. After cooling, 20 mL of a 1 M phosphate buffer (pH 7.0), 5 g of pancreatin, and 50 mg of thymol crystals, included to prevent microbial growth, were added to the suspension. This mixture was incubated at 37°C overnight with occasional stirring. The precipitate was collected via centrifugation (10,000 g, 10 min, 20°C), washed twice with 500 mL of distilled water, and dried overnight at 105°C. The samples were powdered using a mill mixer and filtrated through a 140 mesh sieve. The resulting powder was employed as the insoluble fiber rich fraction in BLP. The insoluble fiber rich fraction was used to obtain the results shown in Figures 4, 5, and 6. The total dietary fiber contents of BLP and the insoluble fiber rich fraction are described in Table 2.

### 2.5. Determination of Dietary Fiber Content

 The total dietary fiber (TDF) content was analyzed, based on AOAC methods (985.29) [14]. Dietary fiber composition, soluble dietary fiber (SDF), cellulose, hemicelluloses, and lignin were analyzed based on the Southgate method [15]. 

### 2.6. Determination of Bound Water in Fibers

Bound water in the fibers was measured based on a centrifugation method [13]. Thirty milligrams of sample was transferred to 1.5 mL polypropylene centrifuge tubes (*n* = 3), and 1 mL of distilled water was added to each tube. Tubes were shaken by hand and soaked for 30 minutes at 37°C. The tubes were then centrifuged at 2,000 g for 10 minutes at 4°C, free water was eliminated using decantation and wiping with kimwipes, and each tube was weighed. The amount of water bound to each sample was calculated by subtracting the weight prior to water treatment, and was expressed on a total dietary fiber amount basis.

### 2.7. Determination of Glucose Adsorption to Fibers

Fifty milligrams of sample was transferred to 1.5 mL polypropylene centrifuge tubes, and 1 mL of a glucose solution was added to each tube (*n* = 4). Each tube was then shaken via vortex and soaked for 30 minutes at 37°C. After another round of shaking, tubes were centrifuged at 13,000 rpm for 5 minutes at 20°C, and the glucose concentrations of the resulting supernatants were measured using a commercial kit (Glu-CII, Wako Pure Chemical Industries, Ltd., Osaka, Japan).

### 2.8. Animal Study

Animal experiments in the present study were approved by the Ethical Committee of TOYO SHINYAKU Co., Ltd. and the Animal Research Committee of Fukuoka Women's University (Permission nos., 006-1165 and 24-1). The oral sucrose tolerance test was conducted at Toyo Shinyaku Co., Ltd. (Saga, Japan), while the remaining animal experiments were conducted at the Fukuoka Women's University (Fukuoka, Japan). All study designs complied with the Guidelines for the Care and Use of Experimental Animals of the Japanese Association for Laboratory Animal Science. 

#### 2.8.1. Oral Sucrose Tolerance Test

*Dose Finding Study*. Six-week-old male Sprague-Dawley (SD) rats for the oral sucrose tolerance test were purchased from Kyudo Co., Ltd. (Saga, Japan). Rats were housed (2-3 per cage) in polycarbonate cages (W260 × D420 × H180 mm) (Clea Japan, Inc., Tokyo, Japan) in a 1-week quarantine and acclimation period. The animal room was maintained under the following constant conditions: room temperature: 24 ± 4°C; relative humidity: 30–70%; and light term: 12 h (8:00 AM to 8:00 PM). Animals were allowed free access to solid diet MF (Oriental Yeast Co., Ltd., Tokyo, Japan) and tap water.

Before the oral sucrose tolerance test, the plasma glucose concentrations of all rats were measured using a blood glucose test meter, GluTestAce (Sanwa Kagaku Kenkyusho Co., Ltd., Aichi, Japan). Eight-week-old 18 male SD rats were randomly divided into 3 groups with approximately equal plasma glucose concentrations. Each group was administered by oral gavage 2,000 mg/kg of sucrose (Nosan Co., Ltd., Kanagawa, Japan) with no BLP (control; *n* = 6), 500 mg/kg of BLP (low dose; *n* = 6), or 1,500 mg/kg of BLP (high dose; *n* = 6) after more than 16 hours of fasting, respectively.

*Fractionating Study*. Seven-week-old 56 male SD rats were randomly divided into 4 groups with approximately equal plasma glucose concentrations. Each group was administered by oral gavage 2,000 mg/kg of sucrose (Clea Japan, Inc., Tokyo, Japan) with no BLP (control, *n* = 14), 1,500 mg/kg of BLP (BLP, *n* = 14), 385 mg/kg of the soluble fraction of BLP (soluble fraction, *n* = 14), or 1,100 mg/kg of the insoluble fraction of BLP (insoluble fraction, *n* = 14), each of which was administered after a fasting period of more than 16 hours. The doses for the soluble and insoluble fractions were calculated according to individual yield. Blood samples were collected from the tail, and plasma glucose concentrations after 0 (before the administration), 30, 60, and 90 minutes were measured using a blood glucose test meter, GluTestAce (Sanwa Kagaku Kenkyusho Co., Ltd., Aichi, Japan). 

#### 2.8.2. Measurement of Digesta Viscosity

Six-week-old male Wistar rats were purchased from Japan SLC, Inc. (Shizuoka, Japan) and housed individually in cages. The animal room was maintained under the following constant conditions: room temperature: 24 ± 4°C; relative humidity: 30–70%; and light term: 12 h (9:00 PM to 9:00 AM). Animals were allowed free access to solid diet MF (Oriental Yeast Co., Ltd., Tokyo, Japan) and distilled water in a 3-day quarantine and acclimation period.

Six-week-old 9 male Wistar rats were randomly divided into 3 groups with approximately equal body weight. Rats were allowed free access to an AIN76 based diet without cellulose (control, *n* = 3), with 112 g/kg of BLP (BLP, *n* = 3), or with 69 g/kg of the insoluble fiber rich fraction (Insoluble fiber rich fraction, *n* = 3) for 3 days, respectively (Table 3). One hundred twelve g/kg of BLP and 69 g/kg of insoluble fiber rich fraction was equivalent to 50 g/kg of total dietary fiber. All animals were exsanguinated after 3 hours from the start of that dark period on the third day, and the gastric, small intestinal, and cecal contents were collected. The coefficients of viscosity of the gastric, small intestinal, and cecal contents were measured using a digital cone-plate viscometer (HBDV-1 Prime) with a CPE-51 spindle cone (Brookfield Engineering Laboratories, Massachusetts, USA). 

#### 2.8.3. Artificial Digesta for Infusion into the Small Intestine

We prepared 3 types of artificial digesta containing carboxymethyl cellulose (Nacalai Tesque, Inc., Kyoto, Japan) and 46 g/L of D-glucose in distilled water either containing no additives (control group), or supplemented with BLP (BLP group) or the insoluble fiber rich fraction (insoluble fiber rich fraction group). The total amount of D-glucose was compensated according to the density of each artificial digesta (control group, 1.017 g/mL; BLP group, 1.028 g/mL; insoluble fiber rich fraction group, 1.038 g/mL) to yield the same concentration in all artificial digesta. Specific details of the compounding ratio for each of the artificial digesta are as follows: control: 43 g/L carboxymethyl cellulose and 46 g/L D-glucose; BLP: 38 g/L carboxymethyl cellulose, 46 g/L D-glucose, and 123 g/L BLP; and insoluble fiber: 40 g/L carboxymethyl cellulose, 46 g/L D-glucose, and 79 g/L of the insoluble fiber rich fraction. The viscosity of the small intestinal digesta of rats on AIN-76 based diets without cellulose was in accord with that of the artificial digesta containing no fiber (control group). 

#### 2.8.4. Catheterization of the Small Intestine

Eleven six-week-old male Wistar rats were randomly divided into 3 groups with approximately equal plasma glucose concentrations. Three types of artificial digesta were, respectively, infused into the duodenum via an intestinal catheter, in order to eliminate the effects of gastric emptying (control and insoluble fiber rich fraction, *n* = 4; BLP, *n* = 3). The rats were anesthetized *via* isoflurane inhalation through spontaneous respiration and maintained under anesthesia throughout the experiment, after 24 hours of food deprivation. *Via* midline laparotomy, a 5 mm incision was made on the greater curvature of the stomach after ensuring hemostasis by ligation of the blood vessels on the stomach wall. A 40 mm-long, small-bore silicon tube (i.d., 1.5 mm; o.d., 2.5 mm) was connected to a 1.2 m silicon tube with a slightly larger bore (i.d., 2.0 mm; o.d., 4.0 mm). The free end of the small-bore silicon tube was inserted through the incision in the stomach wall and into the duodenum through the pyloric sphincter, so that roughly 10 mm of the tube was placed in the duodenum. To eliminate regurgitation of the artificial digesta, the tube was then fastened in the pylorus using 6–0 nylon monofilament on a curved atraumatic needle (1/2-circle, 14 mm; Natsume Seisakusyo Co. Ltd.) and avoiding the larger blood vessels of the stomach wall. The other end of the silicone tube was exteriorized through the laparotomy. The incisions in the stomach and abdominal wall were closed with interrupted 4–0 nylon monofilament sutures (Natsume Seisakusyo Co. Ltd.).

We infused the artificial digesta into the duodenum for 5 min at a rate of 0.4 mL/min using a syringe pump (MSP-DT2, As One). The amounts of artificial digesta infused in the control, BLP, and insoluble fiber rich fraction groups did not differ between groups (*P* = 0.2; control: 2.19 ± 0.06 mL; BLP: 1.96 ± 0.06 mL; and insoluble fiber rich fraction: 1.83 ± 0.17 mL).

#### 2.8.5. Catheterization Test

Blood was collected from the caudal vein at 0, 15, 30, 45, 60, 90, and 120 min after the start of artificial digesta infusion (postinfusion), using a heparin-treated capillary tube. The capillary tube was centrifuged to obtain plasma for measurement of the plasma glucose concentration (1,500 g, 15 min). The plasma glucose concentrations were measured using a commercial kit (Glu-CII, Wako Pure Chemical Industries, Ltd., Osaka, Japan). For 0, 15, 30, 45, and 60 min, plasma insulin concentrations were also measured using a Rat Insulin ELISA Kit (Morinaga Institute of Biological Science, Inc.).

### 2.9. Clinical Study

 The clinical study was approved by the Institutional Review Board of the University of Nagasaki, in accordance with ethical standards established in the Helsinki Declaration, and informed consent was obtained from all subjects. This study was conducted at the University of Nagasaki (Nagasaki, Japan). 

#### 2.9.1. Subjects

Candidate subjects were male and female healthy volunteers, aged from 20 to 65 years, in the University of Nagasaki (Nagasaki, Japan), who met the following selection criteria:

1. are in good health;
2. are able to abstain from drinking from the day prior to the examination;
3. show a stable increase in blood glucose concentration after a meal.

Regarding criterion number 3, a preliminary examination (screening) was performed on all candidates. The study enrolled 36 candidates as test subjects, none of whom fell under any of the following exclusion criteria:

1. have no custom of eating breakfast;
2. are taking drugs that might affect blood glucose concentration;
3. are taking supplements or functional foods that might affect blood glucose concentration;
4. are diabetic or undergoing diabetes treatment;
5. have serious complications, or have contracted a disease that requires urgent remedy;
6. have a chronic sickness and is taking drugs for that sickness;
7. have a disease or history of operations in the digestive system;
8. have drug or alcohol dependency in the history of a present disease or in their medical history;
9. are in pregnancy or lactation, or have plans to become pregnant during the study;
10. are participating in other clinical studies, taking drugs, or applying cosmetics or drugs to the skin;
11. are judged to be unsuitable test subjects by the study director.

No subject was dropped during the study period. Three of 36 subjects were excluded due to not taking uniform dinner. The general characteristics of the 33 subjects were as follows: age (24.6 ± 8.9 y.o.), height (160.6 ± 6.8 cm), body weight (52.7 ± 7.7 kg), BMI (20.3 ± 2.1), and gender (4 males, 29 females).

#### 2.9.2. Test Foods

We used 1.5 g of BLP (TDF; 669 mg) and a placebo food which had texture, color, and taste similar to water-suspended BLP. BLP and the placebo food were, respectively, suspended in 100 mL of distilled water just before testing. 

#### 2.9.3. Study Design

A randomized, double-blind, placebo-controlled crossover trial was conducted to investigate the effects of BLP on postprandial blood glucose level. Subjects were randomly assigned to an active/placebo sequence (*n* = 18) or a placebo/active sequence (*n* = 18). BLP and placebo were administrated in the morning with an administration interval of at least 1 week, as a washout period. Uniform dinner was prepared for all subjects, who were instructed to take the dinner before 21:00 on the day before administration (for a 12 hour overnight fast) and were also restricted from consuming alcohol on the day prior to administration. All subjects arrived at the university in the morning and completed a small questionnaire regarding their last dinner, amount of alcohol, and physical condition. Each test food was served with 200 g of warmed steamed rice (carbohydrates, 68.0 g). The subjects were required to finish consumption within 10 min, at a comfortable pace. 

#### 2.9.4. Blood Glucose Measurements

After resting for 15 min, fasting blood glucose was measured just before consumption of the test food with rice. Further blood samples were taken at 30, 60, and 120 min after the subjects had begun eating. For this purpose, a blood droplet from a subject finger was drawn into a heparinized hematocrit capillary tube (Terumo Co., Tokyo, Japan) via capillary action. Glucose was measured by a commercial kit (Glu-CII, Wako Pure Chemical Industries, Ltd., Osaka, Japan). 

### 2.10. Statistics

 Results were expressed as mean ± SEM. In the oral sucrose tolerance test and the artificial digesta infusion test, analysis was undertaken regarding differences in the delta blood glucose levels among the groups and over the time course of the experiment (0–120 min) via 2-way ANOVA [16]. When the interaction between the effects of the dose of BLP or a different fraction and the time course was significant, either Fisher's PLSD (for 3 groups) or Tukey's multiple comparison test (for 4 groups) was used to test differences in delta blood glucose levels observed among the groups at each time [16]. For glucose adsorption to fiber, 1-way ANOVA was used to test the differences in the glucose concentrations in the supernatants of the plain glucose solution and those in the cellulose and insoluble fiber rich fraction suspensions [16]. For the infusion of artificial digesta into the duodenum, 1-way ANOVA was used to test differences in the amounts of artificial digesta infused in the control, BLP, and insoluble fiber rich fraction groups [16]. Regarding measurements of digesta viscosity, as there were no interactions between the shear rate and the coefficient of viscosity of the digesta, we analyzed differences in the coefficients of viscosity of the digesta among the control, BLP, and insoluble fiber groups using ANCOVA (as a covariate: shear rate), with subsequent use of Fisher's PLSD [16]. For the clinical study, we analyzed the differences in the delta blood glucose levels among groups, and over the time course of the experiment (0–120 min), and the glucose levels (high or low reactivity; identification of subjects with higher or lower blood glucose increment between 0 and 30 minutes after rice consumption with placebo) using 3-way ANOVA [16]. When interactions among the effects of BLP, time after administration, and difference between glucose levels in subjects were significant, Tukey multiple comparison test was used to test the differences the delta blood glucose levels among groups, at each time. In all experiments, differences were considered significant when the *P*-value was <0.05.

## 3. Result

### 3.1. Oral Sucrose Tolerance Test (Dose Finding Study)

**Figure 2:**
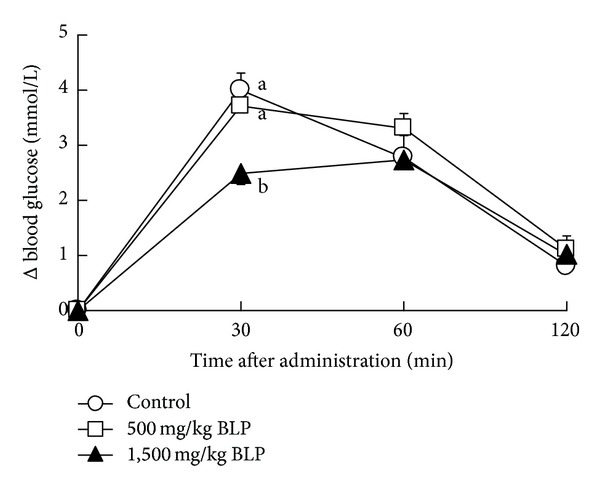
Oral sucrose tolerance test for dose finding of BLP. The delta blood glucose levels in rats at 0–120 minutes after oral administration of 2,000 mg/kg of sucrose with no BLP (control), 500 mg/kg, and 1,500 mg/kg of BLP were measured. Each data point represents mean ± SEM (*n* = 6). A significant interaction was found between the effect of dose and time after administration (*P* < 0.01, 2-way ANOVA). Delta blood glucose levels without a common letter differ (*P* < 0.05, multiple comparisons).

There was a significant interaction between the effects of BLP dose and time after sucrose administration on the blood glucose increment (Figure 2, *P* < 0.01, 2-way ANOVA). There was no significant difference in the blood glucose levels between low BLP dose (500 mg/kg) group and control group at 30, 60, and 120 min after administration (Figure 2; 30 min, *P* = 0.5; 60 min, *P* = 0.2; 120 min, *P* = 0.3; Fisher's PLSD). Blood glucose level in the high BLP dose (1,500 mg/kg) group was lower than that of the control group and the low BLP dose (500 mg/kg) group at 30 min after administration (Figure 2; 30 min, *P* < 0.05; Fisher's PLSD). 

### 3.2. Oral Sucrose Tolerance Test Using Rough Fractionated BLP

BLP is mainly composed of cellulose, hemicellulose, and lignin, which are categorized as insoluble fiber but not as soluble fiber (Table 1). The amounts of total dietary fiber of BLP, the insoluble fraction, and the soluble fraction are described in Table 1. The yields of the insoluble and soluble fractions were 73.3% and 25.6%, respectively. As estimated from the yield, the insoluble fiber contained in 1,000 g of BLP (387 g) was mainly separated into the insoluble fraction (383 g, 73.3% of 537 g of insoluble fiber included in 1,000 g of the insoluble fraction). 

**Figure 3:**
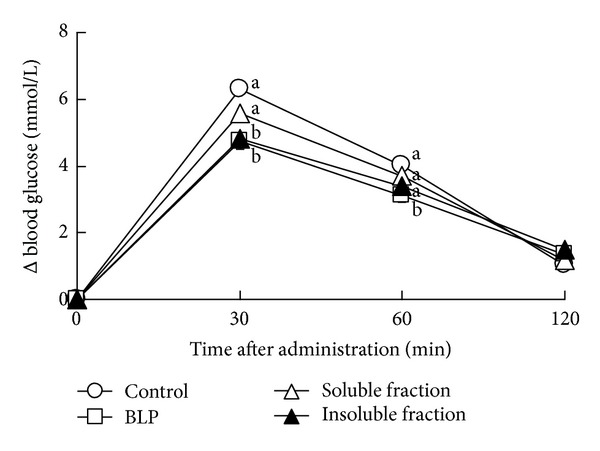
Oral sucrose tolerance test with the water-EtOH soluble and insoluble fractions of BLP. The delta blood glucose levels in rats at 0–120 minutes after oral administration of 2,000 mg/kg of sucrose with no BLP (control), 1,500 mg/kg of BLP, 384 mg/kg of the soluble fraction, and 1,100 mg/kg of the insoluble fraction were measured. Each data point represents mean ± SEM, for *n* = 14 (control, BLP, and soluble fraction) and *n* = 13 (insoluble fraction). A significant interaction was found between the effects of the different fractions and the time after administration (*P* < 0.001, 2-way ANOVA). Delta blood glucose levels without a common letter differ (*P* < 0.05, multiple comparisons).

A significant interaction was found between the effects of different fractions and the time after sucrose administration on the blood glucose increment (Figure 3, *P* < 0.001, 2-way ANOVA). Blood glucose levels in the BLP group were lower than those in the control group at 30 and 60 min after administration (Figure 3, 30 min; *P* < 0.001, 60 min; *P* < 0.05, Tukey multiple comparison test). There was no significant difference in the blood glucose level between the soluble fraction group and control group at 30, 60, and 120 min after administration (Figure 3: 30 min, *P* = 0.2; 60 min, *P* = 0.7; 120 min, *P* = 1.0; Tukey multiple comparison test). The blood glucose level in the insoluble fraction group was lower than that of the control group at 30 min after administration (Figure 3; 30 min, *P* < 0.01; Tukey multiple comparison test). 

### 3.3. Total Polyphenols in the Rough Fractionated BLP

The total polyphenols of BLP and the soluble fraction were 4.6 and 20.1 mg/g, respectively, whereas, total polyphenols of the insoluble fraction were not detected. 

### 3.4. Preparation of the Insoluble Fiber Rich Fraction from BLP

The amounts of total dietary fiber and water holding capacity of BLP, the insoluble fiber rich fraction prepared via the EFRF method, and cellulose as a control are shown in Table 2. 

### 3.5. Glucose Adsorption to Fiber

The glucose concentrations in the supernatants of the glucose samples incubated with no fiber, cellulose, and the insoluble fiber rich fraction of BLP did not differ (*P* = 0.9). 

### 3.6. Measurement of Digesta Viscosity

**Figure 4:**
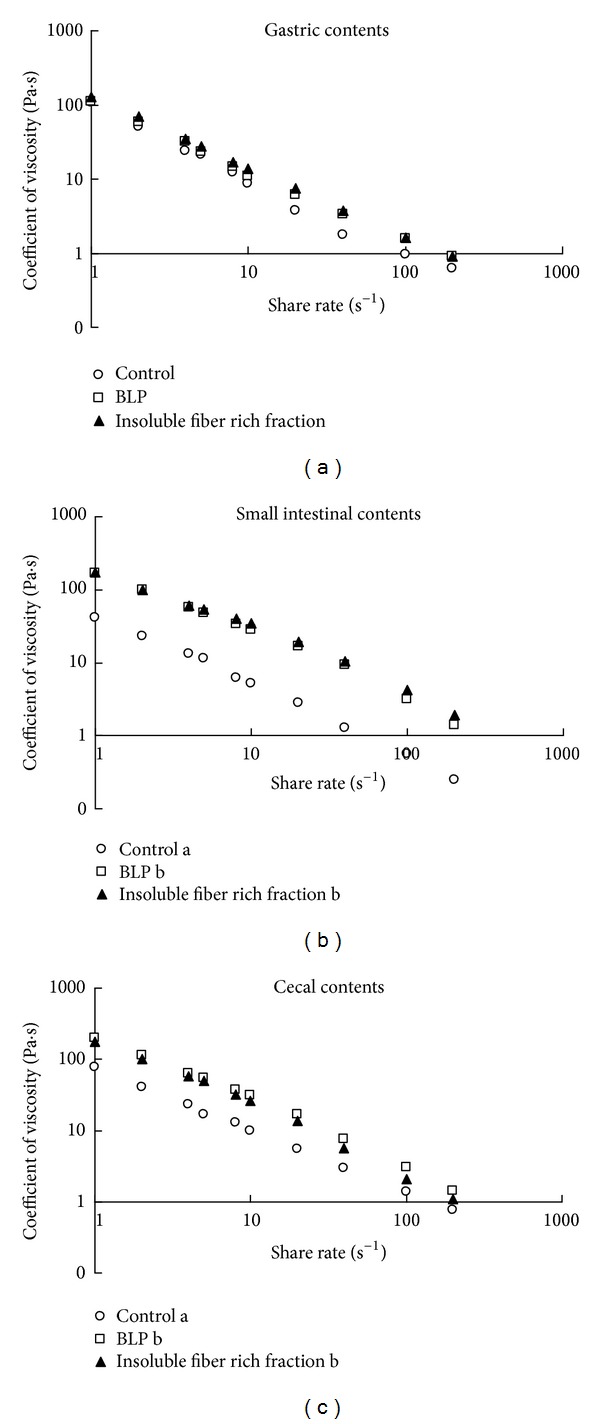
The coefficient of viscosity of (a) gastric contents, (b) small intestinal contents, and (c) cecal contents in rats after administration of no fiber diet (control in the panel), BLP containing diet (BLP in the panel), and BLP-derived insoluble fiber-containing diet (insoluble fiber in the panel). Each data point represents the mean for *n* = 3. Significant differences were found between the coefficients of viscosity of the small intestinal and cecal contents among the groups (*P* < 0.05, ANCOVA). Coefficients of viscosity for gastrointestinal digesta without a common letter differ (*P* < 0.05, multiple comparisons).

There was no significant 2-way interaction between the effect of fiber addition and the shear rate in the gastric, small intestinal, and cecal contents (gastric contents, *P* = 0.9; small intestinal contents, *P* = 0.07; cecal contents, *P* = 0.2; 2-way ANOVA). There were no significant differences in the coefficients of viscosity of the gastric contents among groups (Figure 4(a), gastric contents, *P* = 0.7, ANCOVA). In contrast, significant differences were found between the coefficients of viscosity of the small intestinal and cecal contents among the groups (Figures 4(b) and 4(c); small intestinal contents, *P* < 0.001; cecal contents, *P* < 0.05; ANCOVA). The coefficients of viscosity of the small intestinal and cecal contents in the BLP and insoluble fiber groups were higher than those of the control group (Figures 4(b) and 4(c); small intestinal contents, *P* < 0.01; cecal contents, *P* < 0.05; Fisher's PLSD). 

### 3.7. Catheterization Test of Artificial Digesta with Insoluble Fiber

**Figure 5:**
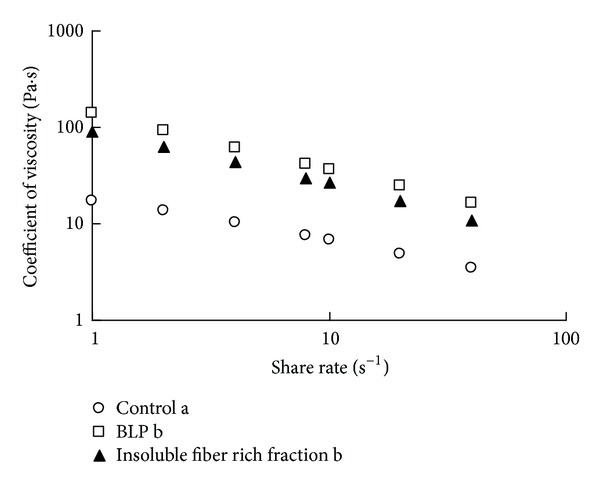
The coefficient of viscosity of control artificial digesta (control), artificial digesta containing BLP (BLP in the panel), and insoluble fiber derived from BLP (insoluble fiber in the panel), before infusion into the small intestine in rats. Significant differences were found between the coefficients of viscosity of the artificial digesta among groups (*P* < 0.01, ANCOVA). The coefficients of viscosity of artificial digesta without a common letter differ (*P* < 0.05, multiple comparisons).

The coefficients of viscosity of the 3 types of artificial digesta are described in Figure 5. There was no significant 2-way interaction between the effect of fiber addition and the shear rate (Figure 5, *P* = 0.1, 2-way ANOVA). There were significant differences in the coefficients of viscosity of the artificial digesta among groups (Figure 5, *P* < 0.01, ANCOVA). The coefficients of viscosities of the artificial digesta in the BLP and insoluble fiber rich fraction groups were higher than those of the control group (Figure 5, *P* < 0.05, Fisher's PLSD). There was no significant difference in the coefficients of viscosity of the artificial digesta, between the BLP and insoluble fiber rich fraction groups (Figure 5, *P* = 0.2, Fisher's PLSD).

**Figure 6:**
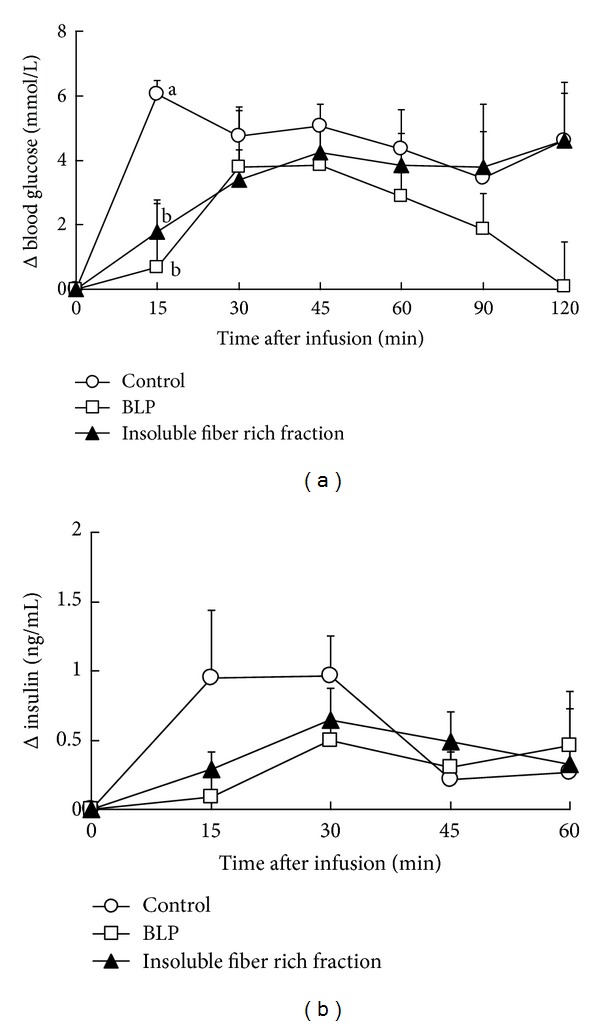
Catheterization test of artificial digesta. (a) The delta blood glucose levels in rats at 0–120 minutes. (b) The delta insulin levels in rats at 0–60 minutes, after small intestinal infusion of the control, BLP-containing (BLP in the panel), and BLP-derived insoluble fiber-containing artificial digesta (insoluble in the panel) were measured. Each data point represents the mean ± SEM for *n* = 4 (control and insoluble fiber) and *n* = 3 (BLP). A significant interaction was found between the effects of addition and time after administration (*P* < 0.05, multiple comparisons). Delta blood glucose levels without a common letter differ (*P* < 0.05, multiple comparisons).

A significant 2-way interaction was found between the effects of addition and time after infusion on the blood glucose increment (Figure 6(a), *P* < 0.05, 2-way ANOVA). The blood glucose levels at 15 minutes after infusion in both the BLP and insoluble fiber groups were lower than those of the control group (Figure 6(a), *P* < 0.05, Fisher's PLSD). Whereas the time to the peak blood glucose level in the control was 15 min, those of the BLP and insoluble fiber groups were later than 15 min. There were no significant differences in the insulin levels (Figure 6(b), *P* = 0.4). One insulin level datum in the BLP group was classified as outlier by the Smirnov-Grubbs test and removed from data analysis.

### 3.8. Clinical Study

**Figure 7:**
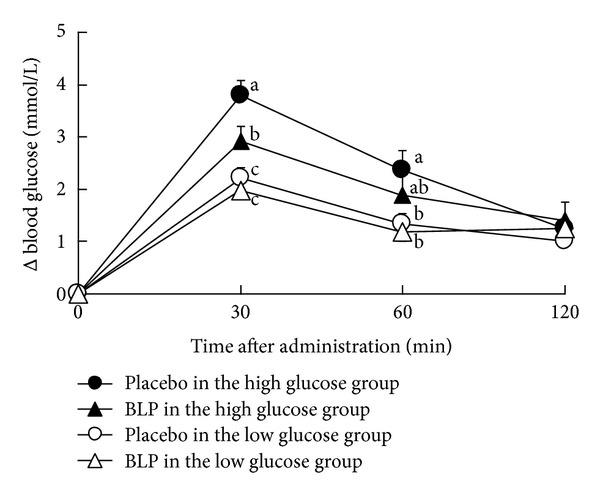
The delta blood glucose levels in humans at 0–120 minutes after administration of 200 g of rice with 0 g (control in the panel) and 1.5 g of BLP (BLP in the panel). The high glucose group (*n* = 12) is composed of subjects whose levels were above the blood glucose level means between 0 and30 minutes after placebo administration. Similarly, the low glucose group (*n* = 21) is composed of those whose levels were below the blood glucose level means between 0 and 30 minutes after placebo administration. Each data point represents mean ± SEM. A significant 3-way interaction was found among the effects of BLP, time after administration, and glucose level (*P* < 0.05, 3-way ANOVA). Delta blood glucose levels without a common letter differ (*P* < 0.05, multiple comparisons).

A significant 3-way interaction was found among the effects of BLP, time after administration and difference in glucose level in subjects (Figure 7, *P* < 0.05, 3-way ANOVA). In the high blood glucose group (*n* = 12), blood glucose level at 30 minutes after administration of BLP was lower than that of placebo (Figure 7, *P* < 0.01, Tukey multiple comparison test). There was no significant difference between BLP and placebo in the low blood glucose group (*n* = 21) at 30 min after administration (Figure 7, *P* = 0.6, Tukey multiple comparison test).

## 4. Discussion

In Japan, the consumption of powdered young barley leaf, known as “Aojiru”, is prevalent. In the present study, we investigated the effects of BLP on postprandial blood glucose, in order to clarify the function of barley leaf powder (BLP). First, we performed an oral sucrose tolerance test to determine the adequate dosage of BLP in rats. BLP, administered at a dose of 1,500 mg/kg, showed lower increment of the postprandial blood glucose level in rats (Figure 2). Next, we performed an oral sucrose tolerance test with flavonoids rich in the rough fraction (soluble fraction) and the insoluble fiber rich rough fraction (insoluble fraction). The insoluble fraction but not the soluble fraction suppressed the increment of the postprandial blood glucose level (Figure 3). Total polyphenols in insoluble fraction of BLP were not detected (see Section 3.3). Effects of polyphenols on the postprandial blood glucose should be ignored in this study. Therefore, we focused on the insoluble fiber component of BLP, in the present study. 

To date, insoluble fibers had not been noted to affect the viscosity of the intestinal contents [17, 18], which should be a suppressor of the increment of the postprandial blood glucose level [19]. Accordingly, previous studies have focused on the suppressive effect of soluble fibers on postprandial blood glucose [20]. However, our previous studies show that insoluble fibers such as cellulose increased the viscosity of the intestinal contents and suppressed the increment of postprandial blood glucose level [9, 11]. The presence of a high viscosity of the intestinal contents due to BLP, which is rich in insoluble fibers (Table 1), might suppress the increment of the postprandial blood glucose level, as noted in previous studies [11].

In this study, both BLP and the insoluble fiber rich fractions were found to increase the viscosity of the small intestinal contents of rats (Figure 4). The higher viscosity obtained with BLP might be due to the higher water holding capacity of BLP fibers (Table 2), as the water holding capacity of insoluble fibers is positively correlated with the viscosity of the intestinal contents [10].  Figure 6 shows that such a high viscosity of the small intestinal contents, as observed with the addition of BLP which has a high water holding capacity, suppressed the increment of the postprandial blood glucose level in rats. Generally, crystalline cellulose, which exhibits a low water holding capacity, suppresses the increment of the blood glucose levels by increasing the viscosity of the small intestinal contents [11]. BLP and the insoluble fiber rich fractions exhibited a much higher water holding capacity than cellulose (Table 2). The suppressive effect of the insoluble fiber in BLP on the increment of blood glucose levels might be much higher than that of cellulose.

Insoluble fibers had been regarded to have the function of binding glucose, referred to as glucose adsorption [21]. We also measured the adsorption of glucose to the insoluble fibers of BLP (see Section 3.5). The adsorption of glucose to cellulose and the insoluble fibers of BLP was not observed in the present study, as in the previous study [11]. Cellulose exhibits high viscosity and suppresses the increment of postprandial blood glucose level and shows no adsorption of glucose [11]. Accordingly, the adsorption of glucose might not be an important function for the suppressive effect of insoluble fibers on postprandial blood glucose.

In the animal experiment involving Figure 6, we eliminated the dilution effect of fibers (see Section 2.8.3). Without the dilution and adsorption (binding) effect, insoluble fibers suppressed the increment of postprandial blood glucose level (Figure 6). The function of the insoluble fibers in BLP involving postprandial blood glucose might be primarily due to high digesta viscosity.

The postprandial blood glucose should depend negatively on the diffusion of glucose in the intestinal contents in the intestinal lumen [19]. Glucose in the intestinal lumen has to be translocated to the epithelium by self-diffusion in the poor mixing environment in the lumen [19]. Such translocation of glucose into the lumen is a rate-limiting factor in the absorption rate of glucose [19]. The lower absorption rate of glucose accompanying high viscosity of the intestinal contents should suppress the increment of the postprandial blood glucose level [22]. We confirmed the lower coefficient of diffusion for glucose in artificial digesta with insoluble fibers (Takahashi et al., unpublished data). Thus, BLP and the insoluble fibers of BLP might decrease the diffusion rate of glucose in the intestinal contents, due to the higher digesta viscosity with BLP and the insoluble fibers of BLP, which then might induce suppression of the increment of postprandial blood glucose level.

Our design was intended to eliminate the effects of gastric emptying and digestion on the postprandial blood glucose in the animal experiment (Figure 6). Gastric emptying and digestion also affect the postprandial blood glucose [23, 24]. Generally, the yield of enzyme products depends negatively on the viscosity of the suspension [25–29]. The presence of a high viscosity of the intestinal contents along with BLP might reduce digestion. Furthermore, insoluble fibers such as cellulose slow gastric emptying [30, 31]. BLP might also decrease gastric emptying. Low gastric emptying and/or digestion rates accompanying the consumption of BLP should suppress the increment of postprandial blood glucose level, as in previous studies [23, 24]. It is possible that BLP might suppress the increment of postprandial blood glucose level not only by decreasing the absorption of glucose in the small intestine but also by slowing gastric emptying and digestion.

In the present study, we conducted a clinical study to investigate the effect of BLP on postprandial blood glucose (Figure 7). It is interesting that BLP suppressed the increment of postprandial blood glucose level in humans, who had higher blood glucose levels after a meal.

In conclusion, consumption of BLP, which possesses high water holding capacity, yields considerably high viscosity of the small intestinal contents in rats. The high viscosity of artificial digesta with BLP and the administration of BLP suppress the increment of postprandial blood glucose both in rats and subjects that had higher postprandial blood glucose, without adsorption of glucose to the insoluble fiber of BLP. BLP might decrease the absorption rate of glucose by decreasing the diffusion rate of glucose in the small intestinal lumen, which should induce lower postprandial blood glucose.

## Figures and Tables

**Table 1:** Dietary fiber composition of BLP, soluble, and insoluble fractions.

	BLP	Soluble fraction	Insoluble fraction
Soluble fiber (g/kg)	11	30	14
Cellulose (g/kg)	209	6	278
Hemicellulose (g/kg)	141	22	198
Lignin (g/kg)	37	ND	47

Total dietary fiber (g/kg)	398	58	537

ND: nondetected.

**Table 2:** Total dietary fiber and water holding capacity of BLP, insoluble fiber rich fraction, and cellulose.

	BLP	Insoluble fiber rich fraction	Cellulose
Total dietary fiber (g/kg)	446*	728*	930
Water holding capacity** (mg/fiber)	9.9 ± 0.2***	9.8 ± 0.4***	3.6 ± 0.1***

*AOAC methods.

**Per 1 g total dietary fiber.

***Mean ± SEM (*n* = 3).

**Table 3:** Diet composition of measurement of digesta viscosity study.

	Control	BLP	Insoluble fiber rich fraction
Casein (g/kg)	200	200	200
Cornstarch (g/kg)	150	150	150
Sucrose (g/kg)	550	438	481
DL-methionine (g/kg)	3.0	3.0	3.0
Corn oil (g/kg)	50	50	50
Choline bitartrate (g/kg)	2.0	2.0	2.0
AIN mineral mixture (g/kg)	35	35	35
AIN vitamin mixture (g/kg)	10	10	10

BLP (g/kg)	—	112	—
Insoluble fiber rich fraction (g/kg)	—	—	69
